# Remarks upon Elephantiasis

**Published:** 1857-07

**Authors:** V. H. Taliaferro

**Affiliations:** Atlanta, Georgia


### ARTICLE II.

*Remarks upon Elephantiasis.* By V. H. TALIAFERRO, M. D., Atlanta, Georgia.

Having had some little experience in the treatment of Elephantiasis, we propose to give you a very short and hastily written article upon that disease. It has received its name from its supposed resemblance to the elephant's leg, and is variously denominated Elephantiasis arabum, Elephantiasis grecorum, Barbadoe's leg, &c., each receiving its appellation from the country in which it is prevalent. Elephantiasis grecorum differs materially from the other varieties of the disease, being described as a tuberculous affection. We propose to treat more particularly of this variety, denominated Elephantiasis arabum. It prevails to a remarkable degree, as an endemic in tropical regions, not being seen elsewhere, only sporadically.

The Etiology, pathology, &c., of the disease seems as yet to have been but little understood. The most generally received opinion is, however, that it commences with inflammation of the lymphatics of the part affected. Our observation does not lead us to the belief, that it is ordinary inflammation of the lymphatic vessels ; but, that it is a specific disease, predisposed to, by peculiarity of constitution. If it is ordinary inflammation, we are, at least, unable to relieve it with remedies usually applied to inflammatory affections. The disease most frequently developes itself in the lower extremities, the leg from the foot or ankle, being its most usual seat.

Next in frequency, is the scrotum and superior extremities. No part of the body is supposed to be exempt from the disease.

In its commencement, we have considerable constitutional disturbance of an inflammatory character—the part affected becoming hot, painful and swollen. In two or three days, with or without treatment, these symptoms begin gradually to subside, and, finally, no trace of disease is left, save slight enlargement of the limb, or part affected. Aside from this enlargement, the patient is apparently free from disease, which is certainly not the course of ordinary inflammation of the lymphatics, or other system of vessels. In the course of a month or six weeks, the patient is again seized with a paroxysm, similar, in every particular, to the first, with the exception of the increased enlargement of the affected part. The subject of this disease, as before stated, in the interval of the exacerbations, is apparently free from disease, and hence, the inference of the existence of some specific cause for the recurrence of the paroxysms. These paroxysms do not recur at stated periods, but sooner or later, the patient is subjected to them, usually, some month, six or eight weeks intervening. After each successive paroxysm, the limb becomes more and more enlarged, until finally, we have the enormous distension, characteristic of the disease. The leg sometimes attains such a size as to become a convenient seat for the patient, when he is weary of carrying about his uncouth burthen.

After the disease has progressed for some considerable length of time, the limb becomes excessively hard and unyielding, the skin thick and scaly, and in some places presents a rugged and fissured appearance, and about the foot and ankle, we have the skin presenting a thick velvety appearance. The writer of an article in Dunglison's Cyclopœdia thus speaks of the enlargement: "The writer of this article was requested to visit a woman at Galle, who, although only about 25 years old, had both her inferior extremities so much enlarged and deformed, that the back part of the legs rested upon the ground, and projected behind the heel, about 9 or 10 inches. The fissures and interspaces between the projecting and pendulous protuberances had ulcerated, and were discharging a most offensive sanies." The disease is said to be sometimes exceedingly migratory, affecting various parts of the body in quick succession.

The writer of the same article referred to says, "Dr. Musgrave thinks this disease should be termed the migratory inflammation of the lymphatic system. Whatever may be its original seat, the patient is never secure, while the constitutional disturbance exists, from a sudden retrocession to some vital organ, I have seen it in the same case transmitted from the scrotum to the head, from thence, after some hours, descend with the rapidity of lightning to the abdomen; again migrate to the chest, to return, perhaps, to the encephalon, and prove fatal there; or, under more favorable circumstances, resume its comparatively harmless situation, and run its subsequent course, as if nothing untoward had occurred."

The increased enlargement left after each paroxysm is, doubtless, owing to an effusion of coagulable lymph into the cellular tissue, which renders the limb excessively hard; and from the thickened, rugged, and fissured condition of the skin, almost insensible to external agents. The reason this effusion is not more readily taken up by the absorbents, is probably in consequence of its coagulation, and it may be, from this fact, that ulceration so rarely occurs.

As to the cause of this affection, but little is known. It is, by some, supposed to be produced by the heat of a tropical sun, and from the fact that it prevails endemically, only in tropical regions, the supposition is reasonable, that it acts either as an exciting or predisposing cause.

We think, however, the most plausible theory to be, that the predisposition exists in a peculiar specific diathesis, and its exciting cause, when prevailing as an endemic, tropical heat. We believe that other causes, as injuries, &c., may *excite* the disease.

As before stated, we believe the predisposing cause to be in constitutional diathesis.

The scrofulous and tuberculous diathesis developes scrofula and consumption in a majority of those exposed for a length of time, to exciting causes. Few escape, who are predisposed to consumption, when exposed to an extreme degree of cold. In Elephantiasis, the reverse exists as regards temperature, the disease being developed, in a majority of those predisposed, by excessive heat, while sporadic cases only occur when not exposed to the usual exciting cause.

We have no doubt, but that the disease may exist from hereditary predisposition, as almost every member of large families have been known to be affected with the disease. I was once told by a physician of experience and medical attainment, that he had treated the disease in a family in which several members were affected, and did not, at that time, know of its existence elsewhere.

In this connection, we will briefly notice a case, which has for the past few months been submitted to our care. William, negro man, about 35 to 40 years of age, has for the last five or six years, been afflicted with an enlargement of his left leg, which includes the foot and ankle, and extends to the knee joint. The disease commenced in his case, after sustaining a slight injury upon the leg, since which time, according to his own statement, he has been the subject of exacerbations, occurring irregularly, from a month to six or eight weeks intervening. When placed under our care, the enlargement of his leg was enormous, and his foot being implicated in the disease was almost round; the whole presenting really, in many respects, the appearance of the elephant's leg. The skin was rough, rugged and in places deeply fissured. About the foot and ankle it presented a thick velvety appearance. The leg was as hard and unyielding almost, as bone, and its sensibility very greatly obtunded. The patient presented no constitutional disturbance, and was able to do the work of an ordinary hand.

But a few weeks had elapsed, after being submitted to our care, when he was attacked with one of those exacerbations peculiar to the disease, at which time his constitution was greatly disturbed. He had excitement of the circulation, nausea and heat of the skin. The attack came on suddenly and without promontory symptoms. At the time of the exacerbation, his leg was tightly bandaged, and such was the intolerable pain produced by the distension, that it was necessary to remove it. Since that time, the exacerbations continue to recur, every six or eight weeks, but with less severity ; each paroxysm seeming to be milder than the preceding, until now, which has been some four months, he is able to retain the bandage, during those periods, and suffers but slight constitutional derangement. This boy, I have learned, has been subject to an immense amount of suffering, both constitutional and local, during these exacerbations; and at one time his other leg swelled to almost the size of the one diseased, which, however, returned to its normal size, after the subsidence of the excitement, and so far as I am informed, has never before or since been affected.

For two or three years, previous to being placed under our care, this boy had been living upon a farm, and his owner informs me, that save the periods of exacerbations, he did the work of an able hand, carrying about his enormous limb with apparent ease and comfort.

During the past four months, this boy has undergone a vigorous treatment, which consists first, in the use of iodine and mercurial ointment to the limb, once or twice a day, applied with considerable friction. This application is made after the limb has been freely bathed with warm water and soap; he is also directed to use frequent ablutions to the whole body.—Twice per day, morning and night, the roller bandage is applied, from the toes to the knee, with considerable force. He wears a shoe made to fit the foot and ankle closely. This constitutes the amount of local treatment.

The constitutional treatment consists principally in the use of Donovan's arsenical solution, administered in doses of from 15 to 18 drops, three times a day in wine glass of water. This remedy is occasionally intermitted for the use of iodide of potassium.

Rest, we are assured, should be strenuously enjoined, but from circumstances, and the extreme difficulty of confining a well negro, so far as feelings are concerned, it has not been enforced. We are at this time much pleased to see that the limb has very much improved, both in reduction of size and in its general appearance. Instead of its being as at first, hard and unyielding, it is now soft and is freely indented, as in anasarca. The skin is now entirely smooth, only at places where it was fissured. From the improvement thus far, we are forced to believe that the limb may be reduced to its natural size.

